# Influence of Hemorrhagic Complications of Pancreatoduodenectomy in Patients with Cancer on Short- and Long-Term Mortality

**DOI:** 10.3390/jcm12082852

**Published:** 2023-04-13

**Authors:** Alexandre Thobie, Fabien Robin, Benjamin Menahem, Jean Lubrano, Karim Boudjema, Arnaud Alves, Olivier Dejardin, Laurent Sulpice

**Affiliations:** 1 Department of Digestive Surgery, University Hospital of Caen, CS 30001, CEDEX 9, 14033 Caen, France; menahem-b@chu-caen.fr (B.M.); lubrano-j@chu-caen.fr (J.L.); alves-a@chu-caen.fr (A.A.); 2 “ANTICIPE” U1086 INSERM, Team Ligue Contre le Cancer, Centre François Baclesse, University of Caen Normandy, 14000 Caen, France; olivier.dejardin@unicaen.fr; 3 Department of Digestive Surgery, University Hospital of Rennes, 35000 Rennes, France; fabien.robin@chu-rennes.fr (F.R.); karim.boudjema@chu-rennes.fr (K.B.); laurent.sulpice@chu-rennes.fr (L.S.); 4 Department of Clinical Research, University Hospital of Caen, 14000 Caen, France

**Keywords:** pancreatoduodenectomy, hemorrhage, survival

## Abstract

Background: With a mortality rate of up to 30%, post-pancreatectomy hemorrhage (PPH) remains a serious complication after pancreatoduodenectomy (PD) for cancer. Little is known about the long-term survival of patients after PPH. This retrospective study aimed to evaluate the impact of PPH on long-term survival after PD. Methods: The study included 830 patients (PPH, n = 101; non-PPH, n = 729) from two centers, who underwent PD for oncological indications. PPH was defined as any bleeding event occurring within 90 days after surgery. A flexible parametric survival model was used to determine the evolution of the risk of death over time. Results: At postoperative day 90, PPH significantly increased the mortality rate (PPH vs. non-PPH: 19.8% vs. 3.7%, *p* < 0.0001) and severe postoperative complication rate (85.1% vs. 14.1%, *p* < 0.0001), and decreased median survival (18.6 months vs. 30.1 months, *p* = 0.0001). PPH was associated with an increased mortality risk until the sixth postoperative month. After this 6-month period, PPH had no more influence on mortality. Conclusions: PPH had a negative impact on the short-term overall survival beyond postoperative day 90 and up to six months after PD. However, compared to non-PPH patients, this adverse event had no impact on mortality after a 6-month period.

## 1. Introduction

Although pancreatic surgery is associated with an increased risk of morbidity and mortality, multidisciplinary care at specialized centers has significantly improved postoperative surgical outcomes [1,2]. Pancreatoduodenectomy (PD) remains a surgically challenging procedure, with a high morbidity rate of 40% [3,4]. Postoperative pancreatic fistula (POPF) is the most frequent complication of PD, with a prevalence of up to 30% depending on the study [5]. POPF-related mortality is 1.0% according to a recent meta-analysis and is associated with the grade of fistula (mortality of Grade C reaches 25.7% [5]). Hemorrhage after PD, or postpancreatectomy hemorrhage (PPH), is the second most serious postoperative complication, which could worsen POPF. This serious complication has a mortality rate of 7.1–23.3% [6,7]. More than 10 years ago, the International Study Group for Pancreatic Surgery (ISGPS) defined PPH as “any postoperative hemorrhagic event after PD, including the severity of the clinical picture and the time of onset” [8]. Despite a prevalence of 2–16% [9,10,11], PPH remains a lethal complication, with mortality of up to 30%, which is known to many digestive surgeons. First, PPH could be life-threatening in the short term. Second, PPH could lead to septic complications in the medium term and delay hospital discharge. Furthermore, the diagnostic and therapeutic management of PPH is complex, and multidisciplinary care, including an interventional radiologist, resuscitator, anesthetist, and gastroenterologist, and prevention of a pancreatic leak is necessary [12]. While the medium- and long-term surgical outcomes of esogastric and colorectal cancer surgery [13,14,15] and pancreatic surgery [16,17] have been investigated, only a few studies have evaluated these parameters for PPH. Pancreatic leak after PD has been reported to be a negative prognostic factor and an independent prognostic marker of disease-free survival [16,18,19]. Hypotheses suggest that severe complications may lead to delayed adjuvant treatment or early recurrence due to local inflammation [20]. The literature is poor concerning the impact of PPH on long-term outcomes after PD for cancer. This bicentric study aimed to assess the impact of PPH on the postoperative survival of cancer patients who underwent PD.

## 2. Materials and Methods

### 2.1. Population

In this retrospective study, we identified all consecutive patients, >18 years old, who underwent PD for oncological indications at two university hospital centers (University Hospital of Caen and University Hospital of Rennes) from the hospital databases between 1 January 2005 and 31 December 2017, and examined the data retrospectively. According to hospital volume, these two university hospital centers were defined as high-volume centers for pancreatic surgery (≥20 resections annually) [21]. Oncological indications included pancreatic ductal adenocarcinoma (PDAC), common bile duct carcinoma (CBDC), neuroendocrine tumors, duodenal adenocarcinoma, ampullary cancers, malignant intraductal papillary mucinous neoplasms, kidney cancers, and metastasis. Other non-oncological indications, such as chronic pancreatitis, pancreatic pseudocyst, and duodenal polyps, were excluded. This study was approved by the Institutional Ethics Committees of the two participating centers (approval number: 1776).

### 2.2. Exclusion Criteria

Patients with the following criteria were excluded: age < 18 years old, non-oncologic indication (chronic pancreatitis, benign pancreatic cyst), other pancreatic surgery besides PD.

### 2.3. PPH Definition

PPH was defined as any bleeding event occurring within 90 days after surgery, according to the ISGPS classification, with three parameters: time of onset (early, ≤24 h after the end of the index operation; or late, >24 h), location (intraluminal, within the digestive tract; or extraluminal, within the peritoneal cavity or drainages), and severity (moderate or severe) [8]. Three different grades of PPH (grades A, B, and C) were defined according to the onset time, location, and severity of bleeding, as well as its clinical impact. The population was divided into 2 groups: PPH and non-PPH groups.

### 2.4. Clinical Variables

The following demographic characteristics of the patients were collected: age, sex, weight at time of surgery, height, body mass index (BMI), body surface area, American Society of Anesthesiologists (ASA) score, comorbidities (including diabetes), and neurological, cardiological, and pneumological histories. The characteristics of pancreatic pathology comprised indication, preoperative endoscopic or radiological biliary drainage, and occurrence of cholangitis with or without acute pancreatitis. Information on neoadjuvant treatment was also recorded.

### 2.5. Intraoperative and Postoperative Variables

The following findings of operative reports and anesthetic records were collected: surgical approach (laparotomy, laparoscopy, or robot), requirement of conversion in cases of laparoscopic or robotic approach, operating time (in minutes), intraoperative hemorrhage loss (in mL), requirement for intraoperative blood transfusion, number of transfusion units of red blood cells, removal of contiguous organs, vascular removal (venous, arterial, and type of removal), vascular reconstruction, requirement for portal clamping and its duration (in minutes), and pancreatic anastomosis type (pancreatojejunostomy or pancreatogastrostomy). Data on the macroscopic quality of the pancreas (soft or hard aspect) were evaluated by a surgeon. Drainage data at the end of surgery were also collected.

Postoperative data included the total length of stay (LOS), hospitalization in the intensive care unit (in days), morbidity at postoperative day 90 according to the Clavien–Dindo classification [22], and mortality at postoperative day 90. Data on surgical complications included hemorrhagic complication and its grade (according to the ISGPS classification) [8] and biliary fistula occurrence. Furthermore, variables of digestive fistula, POPF and its grade (according to the International Study Group for Pancreatic Fistula classification) [23], the presence of intra-abdominal collections on postoperative computed tomography scan, medical complications (neurological, cardiological, nephrological, and septic), and reintervention requirement were recorded.

Concerning the perioperative management of patients undergoing PD, somatostatin analogues were started at a dose of 300 mg/day in the operating theater. On leaving the operating theater, a nasogastric tube was left in place until transit was resumed; drainage of the pancreatic anastomosis was carried out using a non-aspirating blade with systematic amylase measurement on the blade and in the blood from the 3rd postoperative day. The patient was mobilized from the first postoperative day in order to accelerate the resumption of transit.

### 2.6. Histopathological Data

The following pathological findings were collected: histological type, tumor size, number of nodes invaded and removed, and invaded R0/R1 margins. Survival data, including the last follow-up date, clinical status at the last follow-up (alive/deceased), and death date, were also collected.

### 2.7. Statistical Analysis

Overall survival was calculated as the time from the initial diagnosis to the death date or last follow-up date. The end of the study period was 30 April 2020, with no patients lost to follow-up. Median survival rates were calculated using the Kaplan–Meier method. A non-parametric log-rank test was used to compare survival curves.

A flexible parametric survival model (Royston–Parmar model) was used to elucidate the evolution of risk of death over time. While the Cox logistic regression model is commonly used to study survival, this method does not account for the evolution of baseline mortality risk over time, which could be achieved with a flexible parametric survival model; therefore, the flexible parametric Royston–Parmar model was used to determine the survival probability at the third postoperative year as a function of PPH and the evolution of mortality risk over time [24]. The stpm2 procedure on the Stata statistical software was used for this analysis [25]. The Stata software (version 13.1, StataCorp LLC, College Station, TX, USA) was used for the statistical analyses. A value of 0.05 was set for significance.

Survival analyses were performed in the short term (6 months postoperatively) and in the long term (after exclusion of patients who died within 6 months postoperatively). Survival analyses were not stratified according to histological subgroups due to small numbers within each subtype.

## 3. Results

### 3.1. Perioperative Characteristics

#### 3.1.1. Clinical and Operative Characteristics

A total of 830 consecutive patients (PPH, n = 101; non-PPH, n = 729) were included in this study. Among these patients, 59.3% were men and 40.7% were women. The average age of the patients was 65.5 ± 10.9 years (Table 1). The number of women was significantly higher in the non-PPH group than in the PPH group (42.0% vs. 31.7%, *p* = 0.048). The incidence of preoperative cholangitis was significantly higher in PPH patients than in non-PPH patients (7.1% vs. 2.2%, *p* = 0.006). No significant differences were noted in age, ASA score, mean BMI or BSA comorbidities of diabetes mellitus or cardiovascular diseases, and neoadjuvant treatment. There were significantly more patients with cardiovascular diseases and fewer patients with preoperative biliary drainage in the PPH subgroup with grade C than in the non-PPH subgroup (*p* = 0.004 and *p* = 0.03, respectively). There were twice as many patients with neoadjuvant chemotherapy in the PPH subgroup with grade C as that in the non-PPH group; however, the difference was not statistically significant (7.8% vs. 14.0%, *p* = 0.2).

#### 3.1.2. Operative Outcomes

Compared with non-PHH patients, PPH patients were significantly associated with a soft pancreatic texture (PPH vs. non-PPH: 75.0% vs. 49.9%, *p* < 0.0001) and a longer operating time (334.5 vs. 311.4 min, *p* = 0.04) (Table 1). No significant differences were noted in vascular resection, intraoperative transfusion, and the average number of transfused red blood cell units between the two groups.

#### 3.1.3. Surgical Outcomes

On postoperative day 90, the overall mortality rate of all patients who underwent PD was 5.7%. The mortality and severe morbidity (grade ≥ IIIb) rates of the PPH group in cases of PPH were four (19.8% vs. 3.7%, *p* < 0.0001) and six (85.1% vs. 17.1%, *p* < 0.0001) times higher than those of the non-PPH group, respectively. PPH occurrence was significantly correlated with an increased prevalence of pancreatic fistula, biliary fistula, gastro-jejunal fistula, and intra-abdominal collections (Table 2). There were three PPH patients with grade A (3.0%), 48 with grade B (47.5%), and 50 with grade C (49.5%). The characteristics of each PPH grade are reported in Table S1. Total LOS was higher in the PPH group than in the non-PPH group (28.8 vs. 17.4 days, *p* < 0.0001). In addition, the number of patients admitted to the intensive care unit (60.3% vs. 18.4%) and the LOS in the intensive care unit (10.2 vs. 1.6 days, *p* < 0.0001) were higher in the PPH group than in the non-PPH group (Table 2). Time of onset for grade B PPH and grade C PPH was 8.9 and 13.2 days (Supplementary Table S1). In grade B PPH, 41.8% of patients needed a reintervention to control bleeding, versus 72.0% in grade C PPH (*p* = 0.005). Detailed results on the onset, locations, and management of PPH are shown in Supplementary Table S1.

#### 3.1.4. Oncological Outcomes

Oncological outcomes are presented in Table 3. No significant differences were noted in surgical indication (*p* = 0.2), tumor size (PPH vs. non-PPH: 2.7 vs. 3 cm, *p* = 0.3), removed lymph nodes (15.7 vs. 17.8, *p* = 0.07), invaded lymph nodes (1.5 vs. 2, *p* = 0.8), or invaded margins (16.3% vs. 15.2%, *p* = 0.8) between the two groups. For PDAC, no significant difference in the AJCC (American Joint Committee on Cancer) 2017 stage was observed (*p* = 0.4).

### 3.2. Survival Analysis

**Figure 1:**
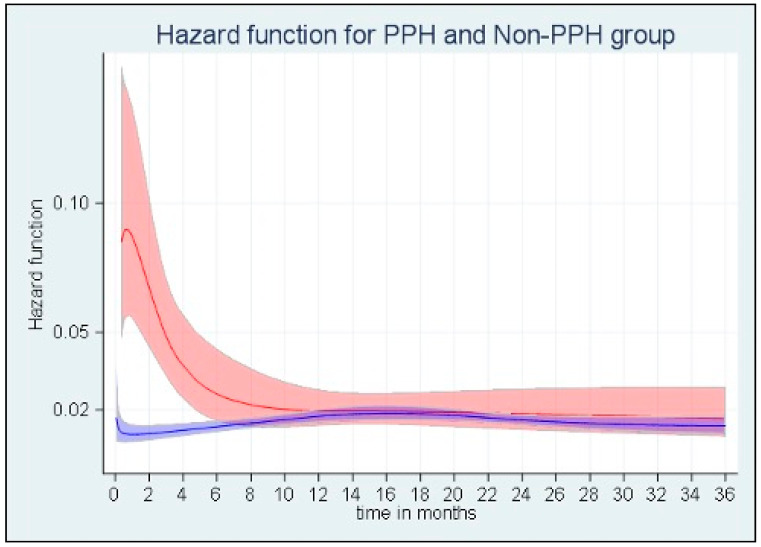
The functions of mortality risk over time in the PPH (red, n = 101) and non-PPH (blue, n = 729) groups in the flexible parametric survival analysis.

The median survival times were 30.1 months (28.2–35.0 months) and 18.6 months (11.4–24.0 months) in the non-PPH and PPH groups, respectively (log-rank test, *p* = 0.0001). Figure 1 shows the evolution of mortality risk over time with a parametric flexible survival model. The mortality risk was significantly higher in PPH patients than in non-PPH patients until six months post-operation. After this 6-month period, the mortality for PPH patients was comparable to non-PPH patients.

Results on short-term and long-term overall survival are presented in Table 4. Short-term overall survival was measured at 6 months post-operation. In the multivariate analysis, PPH (*p* < 0.0001) was a pejorative short-term prognostic factor, especially grades B and C PPH. The other short-term prognostic factors were receipt of adjuvant chemotherapy, resection margin, and indications of resection other than PDAC and CBDC. In long-term overall survival, patients who died before 6 months were excluded from analysis. PPH was no longer a prognostic factor (*p* = 0.2). Advanced age, lymph node invasion, invaded resection margins, and PDAC were pejorative prognostic factors.

## 4. Discussion

Our results highlight that the influence of PPH after cancer resection on mortality is limited to short-term survival. Considering only patients who survived at least 6 months, PPH patients and non-PPH patients had comparable mortality risks. Thus, for these patients, PPH is no longer associated with a pejorative prognosis compared to non-PPH patients. This result could have interesting implications for patients’ psychologic well-being.

Our study highlights the dynamics of mortality associated with PPH. Even though the effect of PPH on mortality was not significant after 6 months, mortality increased immediately during the first month after PD and then decreased quickly until six months post-operation. Thus, mortality risk should be assessed until six months post-PD, rather than until day 90 post-operation, as is common practice. This result should be considered in daily practice for the active follow-up of patients after a PPH.

In this study, the prevalence of PPH after PD was 12.2% [11], consistent with previously published data (2–23%) [10,26,27,28]. In large retrospective studies with all types of pancreatectomies, the prevalence of PPH ranged from 7% to 16% [6,29,30,31,32,33]. However, the inclusion of left and total pancreatectomies tended to underestimate the prevalence of PPH because these procedures have a lower incidence of bleeding events. In fact, in a large cohort of 2429 patients, PD, which represented 60.8% of total pancreatectomies, had a PPH incidence of 8.7%, while distal and total pancreatectomies had an incidence of 4.5% and 8%, respectively [31]. A more recent meta-analysis of 7400 patients showed an incidence of 1.6–12.3% for late PPH after 24 h. However, the incidence of PPH was approximately 3% in studies with a more extended cut-off time at postoperative days 5 or 7 [2]. In a previous study, mortality related to bleeding complications was reported to be 21.5% in all PPH grades, with a range of 3.0–66.7% [26,34,35,36]. In our study, the mortality rate reached 30.7% in PPH patients with grade C, which was comparable to the 35% mortality rate in late PPH patients, as reported in a meta-analysis by Roulin et al. [10]. As described in the literature, grade C PPH occurred later in the second postoperative period and was associated with higher mortality rates than grade B PPH [33,37]. POPF occurred in 56.4% of the PPH group. Yekebas et al. reported 55% of PPH associated with POPF [29]. In our centers, Somatostatin analogs were used during the operative and postoperative periods to prevent POPF and every patient had a passive drainage. Lipase and/or amylase were measured on drainage at postoperative days 1, 3, and 5. A control CT scan was performed at postoperative day 7. There was more preoperative cholangitis in the PPH group than in the non-PPH group (7.1% vs. 2.2%, *p* = 0.006). Preoperative cholangitis has been observed as an independent predictor of POPF, which can lead to PPH [38].

The impact of PPH on medium- and long-term outcomes has been scarcely reported. In pancreatic surgery, the impact of severe complications has mainly been described for pancreatic cancer [39,40,41,42,43,44]. Major complications, such as those with Clavien–Dindo grades III and IV, were pejorative prognostic factors in patients who underwent surgery for borderline or locally advanced carcinoma but not in resectable patients. The absence or delayed completion of chemotherapy might explain the negative impact of PPH on survival and earlier tumor recurrence [45]. Many studies have demonstrated that postoperative complications might influence early cancer recurrence via immunomodulatory mechanisms [17,46,47]. Nathan et al. analyzed surveillance epidemiology and end results program data and highlighted that patients who underwent resection for pancreatic cancer with serious postoperative complications had worse survival outcomes, even if these complications were successfully treated [41]. Furthermore, patients who experienced serious complications had more restricted access to adjuvant chemotherapy. Even after excluding deaths within the first 6 months, serious complications had a negative impact on long-term survival. However, serious complications were defined by the length of hospital stay and differed from the Clavien–Dindo classification. These results are in conflict with our findings. For long-term survival, after the exclusion of deaths within the first 6 months, the was no difference in survival between patients in the PPH group and those in the non-PPH group. Other studies are in agreement with these results [48]. In a recent systematic review on the impact of POPF on long-term outcomes, only one study among sixteen found lower overall survival for patients with POPF [49]. Most of these studies used a Cox regression model, which does not take into account the baseline risk or the variation in mortality risk over time.

Compared with the classical semi-parametric regression analysis (Cox model), statistical analysis using a flexible parametric survival model represents an innovative and optimized approach to assess the change over time in the impact of prognostic factors. One of the main strengths of the present study was the use of a flexible parametric survival model (Royston–Parmar model) to better understand the time-dependent postoperative mortality risk of patients [24]. Survival analysis is usually conducted using a semi-parametric Cox model that poorly reflects the clinical significance of the duration of this postoperative outcome. In contrast, this flexible Royston–Parmar model allowed the evaluation of mortality risk over time to better determine the prognostic utility of different factors. In this regard, the Royston–Parmar model provided information on when and how long a prognostic factor could influence survival. Moreover, HR estimation at all time points, which was not possible using the Cox model, could be achieved with the Royston–Parmar model. The present study showed that PPH had a significant impact on survival until the sixth postoperative month. Beyond this period, the survival curves of patients with and without PPH were similar. While the prognostic factors identified in this study might influence the survival outcomes of patients in the short (postoperative mortality) and medium term, they had little impact on long-term survival, as demonstrated with the Royston–Parmar model. Since most investigations of the impact of complications on survival after PD use semi-parametric models, the application of flexible parametric survival models could allow a better understanding of postoperative outcomes.

This study has some limitations. First, due to the retrospective nature of the study, the broad spectrum of histological characteristics, and the absence of cancer recurrence data, the study results should be interpreted with caution; these limitations could result in bias. Some data were missing. For example, it would have been interesting to evaluate the pancreatic fistula risk score since POPF is associated with PPH [50,51], but the Wirsung diameter was missing in a large part of our database. Second, the study results were derived from two high-volume specialized centers, wherein the volume of patients might have influenced the PD morbidity and mortality, as shown in a recent French multicenter study [21]. Third, no histological difference was noted between the PPH and non-PPH groups in the subgroup analyses of PDAC and CBDC. Fourth, factors contributing to the influence of PPH on survival until the sixth postoperative month were not determined. In this regard, the delay in chemotherapy or the inability to perform adjuvant chemotherapy might be accountable for this observation. PPH could delay access to chemotherapy and decrease disease-free survival. In our study, adjuvant chemotherapy was not a long-term prognostic factor. This could explain the lack of difference in long-term survival between the PPH and non-PPH groups. Even though postoperative complications were the main contributing causes of death for such patients, the accurate cause of death was unknown. It would be of interest to have this information to benefit from a better comprehension of the mortality risk during the first 6 months after PPH events. Unfortunately, patients who died outside the two hospitals included had unknown causes of death. Our main result is that after a 6-month delay from PPH, mortality in excess was comparable in both groups. Finally, data on disease recurrence were not available. Given the aforementioned limitations, future multicenter studies are warranted to validate these results.

## 5. Conclusions

This study demonstrated that PPH worsened medium-term survival in patients undergoing PD but not long-term survival. Therefore, mitigative measures must be integrated into therapeutic decisions after PD. The time window between postoperative day 90 and the sixth months following PPH after PD appeared to be a critical period to improve the survival outcomes of PPH patients. However, the increased mortality risk of PPH patients disappeared after six months, providing some prognostic reassurance for PPH patients with a survival time of at least 6 months. Nevertheless, these observations suggest that the six-month mortality rate should be included in PD outcome assessment.

## Figures and Tables

**Table 1:** Clinical and operative characteristics of the PPH (n = 101) and non-PPH (n = 729) groups (* Chi-squared test, ^?^ Student’s *t*-test). Grade B and C PPH groups are compared to the non-PPH group (with only 3 patients, the grade A PPH group does not appear in the table).

Variables	Total (n = 830)	Non-PPH (n = 729) (87.8%)	PPH (n = 101) (12.2%)	*p* Value *	Grade B PPH (n = 48) (5.8%)	*p* Value *	Grade C PPH (n = 50) (6.0%)	*p* Value *
Sex (M/F)	492 (59.3%)/338 (40.7%)	423(58.0%)/306 (42.0%)	69 (68.3%)/32(31.7%)	0.048	29(60.4%)/19(39.6%)	0.7	37(74.0%)/13(26.0%)	0.03
Mean age (years) (standard deviation)	65.5 +/− 10.9	65.4 +/− 11.0	66.0 +/− 10.3	0.6 ^?^	64.4 +/− 1.7	0.6 ^?^	66.9 +/− 1.1	0.2 ^?^
ASA score				0.6		0.4		0.5
1	134 (16.7%)	121 (17.2%)	13 (13.0%)		7 (14.6%)		5 (10.0%)	
2	508 (63.2%)	443 (62.9%)	65 (65.0%)		32 (66.7%)		32 (64.0%)	
3	158 (19.6%)	137 (19.5%)	21 (21.0%)		8 (16.7%)		12 (24.0%)	
4	5 (0.5%)	3 (0.4%)	0 (0.0%)		1 (2.0%)		0 (0.0%)	
missing	26	25	1		0 (0.0%)		1 (2.0%)	
Mean BMI (kg/m²+/−sd)	24.8 +/− 4.2	24.8 +/− 4.2	25.2 +/− 3.7	0.3 ^?^	25.02 +/− 0.5	0.3 ^?^	25.3 +/− 0.6	0.2 ^?^
Mean body surface (m² +/− sd)	1.83 +/− 0.22	1.82 +/− 0.21	1.87 +/− 0.23	0.05 ^?^	1.86 +/− 0.03	0.08 ^?^	1.87 +/− 0.3	0.08 ^?^
Diabete mellitus	144 (17.4%)	132 (18.2%)	12 (11.9%)	0.1	4 (8.3%)	0.08	8 (16.0%)	0.7
Cardiovascular diseases	333 (40.1%)	285 (39.1%)	48 (47.5%)	0.1	17 (35.4%)	0.6	30 (60.0%)	0.004
Loss of weight	320 (38.8%)	287 (39.7%)	33 (32.7%)	0.2	15 (31.3%)	0.2	16 (32.0%)	0.3
Preoperative acute pancreatitis	115 (13.9%)	102 (14.0%)	13 (12.9%)	0.8	6 (12.5%)	0.8	7 (14.0%)	1
Preoperative cholangitis	23 (2.8%)	16 (2.2%)	7 (7.1%)	0.006	4 (8.5%)	0.01	3 (6.0%)	0.08
Preoperative biliary drainage	265 (32.2%)	238 (33.0%)	27 (26.7%)	0.2	17 (35.4%)	0.8	9 (18.0%)	0.03
Neoadjuvant treatment (chemo/radiochemotherapy)	65 (7.8%)	57 (7.8%)	8 (7.9%)	1	1 (2.1%)	0.3	7 (14.0%)	0.2
Operative characteristics								
Surgical approach				0.2		0.4		0.05
Open	806 (97.1%)	710 (97.4%)	95 (95.0%)		46 (95.8%)		46 (92.0%)	
Laparoscopy/Robotic	22 (2.7%)	19 (2.6%)	6 (5.0%)		2 (4.2%)		4 (8.0%)	
Operative time (min) (mean, CI95%)	314.3 [306.9–321.7]	311.4 [303.5–319.2]	334.5 [313.5–355.5]	0.04 ^?^	348.5 [310.7–386.3]	0.03 ^?^	328.3 [297.0–359.5]	0.3 ^?^
Operative blood transfusion	182 (28.3%)	156 (27.3%)	26 (25.7%)	0.1	13 (28.3%)	0.2	15 (33.0%)	0.5
Mean number of blood units (sd)	3.0 +/− 1.8	3.1 +/− 1.7	2.5 +/− 1.9	0.1 ^?^	2.4 +/− 0.5	0.2 ^?^	2.5 +/− 0.6	0.3 ^?^
Extensive resection (other organs)	77 (9.3%)	68 (9.4%)	9 (8.9%)	0.9	2 (4.2%)	0.2	7 (14.0%)	0.3
Vascular resection	192 (23.1%)	164 (22.5%)	28 (27.7%)	0.2	11 (22.9%)	0.9	16 (32.0%)	0.1
Venous resection	164/192 (85.4%)	140/164 (85.4%)	24/28 (85.7%)	1	9 (18.8%)	0.9	14 (28.0%)	0.1
Arterial resection	26 (13.5%)	20 (12.2%)	6 (21.4%)	0.2	3 (6.3%)	0.2	3 (6.0%)	0.2
Quality of pancreatic (soft)	382 (53.0%)	316 (43.3%)	66 (65.3%)	<0.0001	31 (64.6%)	0.001	34 (68.0%)	0.001
Pancreatic anastomosis				1		0.8		0.7
Pancreatogastrostomy	219 (26.5%)	192 (26.4%)	27 (26.7%)		13 (27.1%)		12 (24.0%)	
Pancreatojejunostomy	582 (70.4%)	512 (70.4%)	71 (70.3%)		32 (66.7%)		37 (74.0%)	
None	26 (3.1%)	23 (3.2%)	3 (3.0%)		3 (6.3%)		1 (2.0%)	

**Table 2:** Morbidity and mortality in the PPH and non-PPH groups (* Chi-squared test, ^?^ Student *t*-test). Grade B and C PPH groups are compared to the non-PPH group (with only 3 patients, the grade A PPH group does not appear in the table).

Variables	Total (n = 830)	Non-PPH (n = 729) (87.8%)	PPH (n = 101) (12.2%)	*p* Value *	Grade B PPH (n = 48) (5.8%)	*p* Value	Grade C PPH (n = 50) (6.0%)	*p* Value
Length of stay (days) (median. range)	18.7 +/− 11.4	17.4 +/− 9.4	28.8 +/− 17.3	<0.0001 ^?^	23.7 +/− 14.5	<0.0001 ^?^	33.9 +/− 18.7	<0.0001 ^?^
Morbidity								
Day 90 mortality	47 (5.7%)	27 (3.7%)	20 (19.8%)	<0.0001	5 (10.4%)	0.02	15 (30.0%)	<0.0001
Day 90 Dindo–Clavien > IIIb	210 (25.4%)	124 (17.1%)	86 (85.1%)	<0.0001	35 (72.9%)	<0.0001	50 (100.0%)	<0.0001
Surgical complications								
Pancreatic fistula (POPF)	245 (29.8%)	188 (26.1%)	57 (56.4%)	<0.0001	23 (47.9%)	0.001	33 (66.0%)	<0.0001
Biliary fistula	42 (5.1%)	27 (3.7%)	15 (14.9%)	<0.0001	5 (10.4%)	0.03	10 (20.0%)	<0.0001
Digestive fistula	14 (1.7%)	5 (0.7%)	9 (9.1%)	<0.0001	4 (8.7%)	<0.0001	5 (10.0%)	<0.0001
Collections	194 (23.4%)	149 (20.4%)	45 (44.6%)	<0.0001	18 (37.5%)	0.005	27 (54.0%)	<0.0001
Delayed gastric emptying	442 (53.3%)	382 (52.4%)	60 (59.4%)	0.2	25 (52.1%)	1	33 (66.0%)	0.06
Medical complications								
Infection	176 (21.5%)	134 (18.7%)	42 (42.4%)	<0.0001	17 (36.2%)	0.003	25 (50.0%)	<0.0001
Pulmonary disease	33 (4.0%)	27 (3.7%)	6 (5.9%)	0.3	2 (4.2%)	0.8	4 (8.0%)	0.1
Heart failure	13 (1.6%)	8 (1.1%)	5 (5.0%)	0.003	1 (2.1%)	0.5	4 (8.0%)	<0.0001

**Table 3:** Histological characteristics of the PPH (n = 101) and non-PPH (n = 729) groups (* Chi-squared test, ^?^ Student *t*-test). Grade B and C PPH groups are compared to the non-PPH group (with only 3 patients, the grade A PPH group does not appear in the table).

Variables	Total (n = 830)	non PPH (n = 729)	PPH (n = 101)	*p* Value *	Grade B PPH (n = 48) (5.8%)	*p* Value	Grade C PPH (n = 50) (6.0%)	*p* Value
Histological type				0.2		0.2		0.4
Pancreatic ductal adenocarcinoma	421 (50.7%)	377 (45.4%)	43 (42.6%)		18 (37.5%)		23 (46.0%)	
Common bile duct carcinoma	110 (12.0%)	88 (12.1%)	22 (21.8%)		12 (25.0%)		9 (18.0%)	
Malignant ampulloma	118 (14.2%)	105 (14.4%)	13 (12.9%)		8 (16.7%)		5 (10.0%)	
Intraductal papillary mucinous neoplasm	65 (7.8%)	58 (7.9%)	8 (7.9%)		5 (10.4%)		3 (6.0%)	
Neuro-endocrine tumor	44 (5.3%)	39 (5.3%)	5 (4.9%)		3 (6.3%)		2 (4.0%)	
Duodenal adenocarcinoma	57 (6.9%)	49 (6.7%)	8 (7.9%)		2 (4.2%)		6 (12.0%)	
Kidney cancer metastasis	15 (1.8%)	13 (1.8%)	2 (2.0%)		0 (0.0%)		2 (4.0%)	
Tumor size (cm) (mean, sd)	2.9 +/− 2.0	3.0 +/− 2.0	2.7 +/− 1.6	0.3 ^?^	2.6 +/− 2.0	0.2 ^?^	2.9 +/− 1.6	0.8 ^?^
Number of lymph nodes removed (mean. sd)	17.5 +/− 10.9	17.8 +/− 10.8	15.7 +/− 11.1	0.07 ^?^	13.7 +/− 7.6	0.013 ^?^	17.6 +/− 13.7	0.9 ^?^
Number of lymph nodes invaded (mean. sd)	1.9 +/− 2.9	2.0 +/− 3.0	1.5 +/− 2.5	0.1 ^?^	1.4 +/− 2.4	0.2 ^?^	1.5 +/− 2.4	0.3 ^?^
Invaded margin (R1)	123 (15.4%)	107 (15.2%)	16 (16.3%)	0.8 ^?^	8 (16.7%)	0.7	8 (16.0%)	0.8
AJCC 2017 Stage for Pancreatic Ductal Adenocarcinoma				0.4		0.5		0.3
IA	20 (4.8%)	16 (4.2%)	4 (9.1%)		1 (5.6%)		2 (8.7%)	
IB	28 (6.7%)	25 (6.6%)	3 (6.8%)		1 (5.6%)		1 (4.3%)	
IIA	75 (17.7%)	66 (17.5%)	9 (20.5%)		3 (16.7%)		6 (26.1%)	
IIB	261 (62.0%)	237 (62.9%)	24 (54.5%)		12 (66.7%)		11 (47.8%)	
III	23 (5.5%)	23 (6.1%)	0 (0.0%)		0 (0.0%)		0 (0.0%)	
Unknown	14 (3.3%)	10 (2.7%)	4 (9.1%)		0 (0.0%)		3 (13.0%)	

**Table 4:** Multivariate flexible survival analyses of short- and long-term survival (with only 3 patients, the grade A PPH group does not appear in the table).

Variables	Short-Term Survival Analyses (until 6 Months) *		Long-Term Survival Analyses (after 6 Months) *	
	HRa ^?^ [95%CI]	*p*	HRa ^?^ [95%CI]	*p*
Sex		0.2		0.9
Male	Ref.		Ref.	
Female	0.68 [0.40–1.17]		0.98 [0.77–1.26]	
Age (years)		0.8		0.003
<60 years	Ref.		Ref.	
60–75 years	0.62 [0.32–1.20]		1.03 [0.75–1.42]	
>75 years	0.83 [0.45–1.54]		1.49 [1.09–2.03]	
ASA score		0.7		0.4
≤2	Ref.		Ref.	
>2	0.92 [0.52–1.62]		1.11 [0.83–1.49]	
Histological type		0.001		0.4
PDAC	Ref.		Ref.	
CBDC	1.25 [0.68–2.32]		0.68 [0.46–0.99]	
Others	0.28 [0.13–0.64]		0.53 [0.38–0.74]	
Lymph node invasion^§^		0.5		0.006
No	Ref.-		Ref.	
Yes	1.12 [0.75–1.95]		1.41 [1.06–1.89]	
Tumor size (TNM classification)		0.06		0.01
≤T2	Ref.		Ref.	
>T2	1.98 [0.99–4.01]		1.40 [1.01–1.97]	
Resection margins		0.009		0.02
Disease-free margins	Ref.		Ref.	
Invaded margins	2.09 [1.17–3.74]		1.43 [1.06–1.94]	
Grade PPH		<0.0001		0.2
Non-PPH	Ref.		Ref.	
PPH B	2.31 [1.06–5.04]		1.23 [0.71–2.14]	
PPH C	5.35 [2.75–10.39]		1.35 [0.76–2.40]	
POPF		0.4		0.6
No POPF	Ref.		Ref.	
POPF	1.47 [0.85–2.52]		1.04 [0.77–1.41]	
Adjuvant chemotherapy		<0.0001		0.17
No	Ref.		Ref.	
Yes	0.13 [0.06–0.28]		1.21 [0.92–1.59]	
Neoadjuvant treatment (Chemo/radio-therapy)		0.6		0.7
No	Ref.		Ref.	
Yes	1.16 [0.45–2.99]		1.06 [0.63–1.78]	

* For short-term survival analyses, end point was 6 months postoperatively; for long-term survival analyses, end point was 36 months postoperatively, and deaths within 6 months were excluded from analyses. ^?^ HRa: adjusted Hazard Ratio.

## Data Availability

The data presented in this study are available on request from the corresponding author. The data are not publicly available due to the decision of the local ethical committee.

## Supplementary Materials

The following are available online at https://www.mdpi.com/article/10.3390/jcm12082852/s1, Table S1. Clinical presentation and therapeutic management of PPH grade B (n = 48) and C (n = 50) (* chi2-test, ^?^ Student *t*-test).
