# Supplementary material for: Influence of Hemorrhagic Complications of Pancreatoduodenectomy in Patients with Cancer on Short- and Long-Term Mortality
Source: J Clin Med. 2023 Apr 13;12(8):2852. doi: 10.3390/jcm12082852 (PMC10143756; doi:10.3390/jcm12082852)
Supplement: Supplementary file 1 [file jcm-12-02852-s001.zip › jcm-2315693-supplementary.pdf]

Variables	Grade B (n=48)	Grade C (n=50)	p value[*]
**Time of onset in days (mean) (standard deviation)**	8,9 +/- 14,8	13,2 +/- 9,5	0,1[?]
**Exteriorisation**	38 (82,0%)	37 (75,8%)	0,6
**Sentinel bleeding**	9 (18,8%)	14 (28,0%)	0,3
Time of onset of sentinel bleeding in days (mean, standard deviation)	7,4 +/- 12,5	11,1 +/- 12,9	0,5[?]
Average time to onset of bleeding (mean, standard deviation)	2,7 +/- 5,4	2,7 +/- 7,9	1[?]
**Blood loss**			
*Mean hemoglobin loss compared to D1**	3,5 +/- 2,2	4,5 +/- 2,3	0,06[?]
**Clinical shock**	16 (33,3%)	44 (88,0%)	**<0,0001**
**Transfusion**	37 (77,1%)	46 (92,0%)	**0,04**
Mean of red blod cell unit (mean, standard deviation)	2,9 +/- 2,1	7,4 +/- 5,5	**<0,0001**[?]
**CT-scan**	30 (62,5%)	31 (62,0%)	1
**Arteriography**	15 (31,3%)	20 (40,0%)	0,4
Embolisation	12 (80,0%)	20 (100,0%)	0,1
Stenting	6 (40,0%)	8 (40,0%)	0,6
**Successful**	13 (86,7%)	13 (65,0%)	0,9
**Reintervention**	21 (41,8%)	36 (72,0%)	**0,005**
**Successful**	18 (85,7%)	31 (86,1%)	1
**Endoscopy**	14 (29,2%)	12 (24,0%)	1
**Recurrence**	13 (27,1%)	17 (34,0%)	0,5
**Location of bleeding***			
Gastroduodenal artery	7 (14.6%)	15 (30.0%)	
Superior mesenteric artery	3 (6.3%)	7 (14.0%)	
Hepatic artery	6 (12.5%)	13 (26.0%)	
Splenic artery	5 (10.4%)	5 (10.0%)	
Pancreaticoduodenal arcade artery	4 (8.3%)	3 (6.0%)	
Pancreatic stump	11 (22.9%)	14 (28.0%)	
Cœliac trunk	0 (0.0%)	1 (2.0%)	
Gastrojejunostomy	8 (16.7%)	6 (12.0%)	
Veinous	5 (10.4%)	9 (18.0%)	
Others (liver, spleen, omentum)	6 (12.5%)	8 (16.0%)	
Location not found	15 (31.3%)	3 (6.0%)	

Supplemental data S1. Clinical presentation and therapeutic management of PPH grade B(n=48) and C (n=50) ([*]chi2-test, [?]Student t-test) (*Some patients may have had multiple bleeding sites).
